# Exenatide Improves Glucose Homeostasis and Prolongs Survival in a Murine Model of Dilated Cardiomyopathy

**DOI:** 10.1371/journal.pone.0017178

**Published:** 2011-02-17

**Authors:** Arpita Kalla Vyas, Kai-Chien Yang, Dennis Woo, Anatoly Tzekov, Attila Kovacs, Patrick Y. Jay, Paul W. Hruz

**Affiliations:** 1 Department of Pediatrics, Washington University School of Medicine, St. Louis, Missouri, United States of America; 2 Department of Internal Medicine, Washington University School of Medicine, St. Louis, Missouri, United States of America; 3 Department of Genetics, Washington University School of Medicine, St. Louis, Missouri, United States of America; 4 Department of Cell Biology and Physiology, Washington University School of Medicine, St. Louis, Missouri, United States of America; University of Bristol, United Kingdom

## Abstract

**Background:**

There is growing awareness of secondary insulin resistance and alterations in myocardial glucose utilization in congestive heart failure. Whether therapies that directly target these changes would be beneficial is unclear. We previously demonstrated that acute blockade of the insulin responsive facilitative glucose transporter GLUT4 precipitates acute decompensated heart failure in mice with advanced dilated cardiomyopathy. Our current objective was to determine whether pharmacologic enhancement of insulin sensitivity and myocardial glucose uptake preserves cardiac function and survival in the setting of primary heart failure.

**Methodology/Principal Findings:**

The GLP-1 agonist exenatide was administered twice daily to a murine model of dilated cardiomyopathy (TG9) starting at 56 days of life. TG9 mice develop congestive heart failure and secondary insulin resistance in a highly predictable manner with death by 12 weeks of age. Glucose homeostasis was assessed by measuring glucose tolerance at 8 and 10 weeks and tissue 2-deoxyglucose uptake at 75 days. Exenatide treatment improved glucose tolerance, myocardial GLUT4 expression and 2-deoxyglucose uptake, cardiac contractility, and survival over control vehicle-treated TG9 mice. Phosphorylation of AMP kinase and AKT was also increased in exenatide-treated animals. Total myocardial GLUT1 levels were not different between groups. Exenatide also abrogated the detrimental effect of the GLUT4 antagonist ritonavir on survival in TG9 mice.

**Conclusion/Significance:**

In heart failure secondary insulin resistance is maladaptive and myocardial glucose uptake is suboptimal. An incretin-based therapy, which addresses these changes, appears beneficial.

## Introduction

Despite significant advances, congestive heart failure remains a major cause of morbidity and mortality. Standard medical therapy for congestive heart failure includes the use of ACE inhibitors, angiotensin receptor antagonists, and β-blockers, which inhibit maladaptive neurohormonal signaling pathways. Insulin resistance is also recognized as a common metabolic response to heart failure [1], [2]. Modulation of the delivery to and expenditure of energy in the heart under situations of acute and chronic stress hence has garnered growing interest [3]. Solid evidence indicates that the failing heart is chronically energy depleted, but the specific contributions of reduced energy supply and increased energy utilization remain incompletely characterized. Thus, drugs that enhance insulin sensitivity, myocardial glucose uptake or both have been proposed as potential therapies in heart failure [4]. Incretin mimetics are a new class of anti-diabetic drugs with pleiotropic effects on insulin and glucagon secretion, gastric emptying, satiety, and peripheral insulin sensitivity [5], [6]. Glucagon-like peptide-1 (GLP-1), administered by continuous subcutaneous infusion has been shown to increase myocardial glucose delivery and improve left ventricular function in patients with heart failure [7]. Studies to date however have not established whether these changes are correlated with increased survival in humans or animal models that have cardiomyopathy as the primary defect. It also remains unclear whether this beneficial effect is mediated through direct effects of incretin hormones on contractile function or changes in myocardial glucose delivery [8]. In clinical studies, the presence of other long-standing environmental risk factors (high fat diet, sedentary lifestyle, smoking) and resulting co-morbidities (obesity, insulin, resistance, atherosclerosis, hypertension) together with the need for concomitant drug therapy in human heart failure patients complicate efforts to directly determine the effects of pharmacologic agents that alter cardiac or systemic glucose homeostasis. We report here the beneficial effects of the GLP-1 agonist exenatide, which can be administered by intermittent subcutaneous injection, on glucose homeostasis, cardiac function and survival in a transgenic mouse model of dilated cardiomyopathy.

## Materials and Methods

### Materials

The GLUT4 antagonist Ritonavir (Norvir) was obtained from Abbott pharmaceuticals (Abbott Park, IL). The GLP-1 agonist Exenatide (Byetta) was obtained from (Lilly, Indianapolis, IN). GLUT4 antibody was custom produced by Invitrogen (Carlsbad, CA). GLUT1 antibody was a gift from Dr Mike Mueckler (Washington University, St Louis, MO). GAPDH monoclonal antibody was purchased from Abcam (Cambridge MA). Anti human/rat/mouse monoclonal pan-AKT antibody and rabbit anti-phospho-AKT antibody were ordered from R& D Systems, Inc (Minneapolis MN). AMPKα (F6) mouse antibody and phospho-AMPKα (Thr 172) antibody were ordered from Cell Signaling (Danvers, MA). Secondary anti-mouse and anti rabbit antibodies were ordered from LI-COR (Lincoln, NE). Unless noted, all other reagents were purchased from Sigma (St. Louis MO).

### Mouse Model

The TG9 dilated cardiomyopathy model was developed by transgenic, high-level cardiac-specific expression of the cre recombinase protein driven by the α-myosin heavy chain promoter, as previously described [9]. The line is maintained in the FVB/N strain background. The characteristic development and progression of dilated cardiomyopathy in this mouse strain has been extensively characterized [9], [10], [11].

### Animal Procedures

All animal experiments were approved by the animal studies committee at Washington University School of Medicine (Protocol 20080183). Mice were housed in the animal facility at Washington University under standard light/dark cycles and fed standard mouse chow diet and water *ad libitum*. For survival studies female TG9 mice were given exenatide injections subcutaneously twice daily (40 µg/kg/day) starting at 56 days of age with age-matched littermate controls receiving an equal volume of normal saline. Beginning at 75 days of age a subgroup of exenatide-treated mice received ritonavir (10 mg/kg) by daily intraperitoneal injection. Animals were closely monitored for activity, respiratory rate, and general signs of distress for at least 90 minutes after each injection.

### Glucose tolerance

Following a 5 hour fast mice in the survival study group were subjected to a 2 hour glucose tolerance test. At time zero 10% dextrose (1 g/kg) was administered by an intraperitoneal injection and blood was sampled from a tail vein at 15 minutes intervals. Blood glucose was immediately determined using an Acenscia Contour glucometer (Bayer Health care LLC, Tarrytown, NY).

### Serum analyses

Serum insulin levels were determined by the DRTC immunoassay core facility at Washington University using a Singulex-based assay (Alameda, CA). Serum triglyceride and cholesterol levels were measured using commercially available kits (catalog numbers 339-10 and 352-20, respectively) from Sigma (St. Louis, MO). Serum NEFA levels were determined using reagents obtained from Wako Chemicals (Richmond, Virginia).

### Myocardial glucose uptake

Relative glucose uptake (R_g_) was assessed by measuring 2-deoxyglucose incorporation in left ventricular myocardium under basal conditions following a 5-hour fast as previously described [10], [12], [13].

### Protein expression

Left ventricular myocardium was harvested from the mice immediately following euthanasia and frozen in liquid nitrogen. Lysates were prepared by homogenization in buffer containing 1% triton X100 in PBS, Sigma protease inhibitor cocktail, sodium vanadate 1 mM, sodium fluoride 50 mM, and sodium pyrophosphate 10 mM. Lysates were kept on ice for 15 minutes and cleared by centrifugation at 15,000 g for 20 minutes at 4 degree Celsius. Protein concentration was determined by the Bradford method (Bio-rad, Hercules, CA). Western blot analysis was performed on 8 µg of total protein per lane using GLUT1 (1∶1000) or GLUT4 (1∶1000) rabbit polyclonal antibody recognizing the C terminus of the transporter, pan-AKT (1∶5000), phospho-AKT (1∶2000), AMPKα (1∶1000) and phospho-AMPKα (1∶1000). GAPDH (1∶5000) was used as the control (used mouse monoclonal antibody). Protein band intensities were quantified using Odyssey infrared imaging system version 3.0 (LI-COR biosciences, Lincoln, NE, USA).

### Brain natriuretic peptide RNA Quantification

RNA was purified and cDNA synthesized from 30 to 50 mg of frozen left ventricular myocardium solubilized in trizol. Total RNA, purified from the homogenate using the manufacturer's instruction, was treated with DNAse, followed by a phenol chloroform extraction. cDNA was synthesized from 1.3 µg of total RNA using Super Script First Strand Synthesis System (Invitrogen) according to the manufacturer's protocol. Quantitative Real Time PCR was then performed using BNP sense (5′ TCACCGCTGG GAGGTCACTC 3′) and antisense (5′ GTGAGGCCTTGGTCCTTCAAG 3′) primer sequences. GAPDH was used as the reference gene and targeted GAP-DH sense (5′ CATCCACTGG TGCTGCCAAG 3′) and antisense (5′ GAGGGAGATGCTCAGTGTTGG 3′) primers. PCR reactions were assembled as follows: Per 50 ul reaction 5 ul 10X KLA pH 7.9 reaction buffer was used (DNA Polymerase Technology Inc.), 10 pM sense and antisense primers, 250 µM dNTPs, 1 mM MgCl_2_, 1.5 M betaine (Sigma B2629), 0.05 µL Cesium Klentaq AC (DNA Polymerase Technology Inc.), 0.167X Cybr Green (Invitrogen S7567), 23.7 uL molecular biology quality water (Sigma W4502). 1 uL of cDNA was used per 50 µL PCR reaction. Cycling conditions consist of 1 time incubation at 60°C for 5 min, 94°C for 30 sec, 60°C 30 sec, 68°C 1 min, 40 cycles. The qPCR was performed on a Stratagene MX3005 qPCR thermal cycler.

### Echocardiography

Transthoracic echocardiographic images were obtained on 70 day old TG9 mice after sedation with Avertin. The Acuson Sequoia 256 echocardiography system with 15 MHz transducer (Acuson corp., Mountain View, CA, USA) was used. Left ventricular chamber dimensions and function were measured by M-mode echocardiography. The internal diameter of the left ventricle was measured in end-diastole and end-systole (LVIDd and LVIDs). Systolic function was quantified by fractional shortening, FS =  (LVIDd -LVIDs)/LVIDd.

## Results

### Glucose homeostasis in TG9 mice

The TG9 mouse develops progressive dilated cardiomyopathy in a highly predictable manner. Cardiomyopathy in TG9 mice can be detected by echocardiography at 6 weeks of age as marked by a steady increase in left ventricular diameter and decrease in contractile function. Decompensated heart failure and death occur between 11-13 weeks of life. In addition, TG9 mice exhibit many of the salient molecular and functional changes observed in human heart failure including elevated brain natriuretic peptide levels and a positive response to beta blockers and ACE inhibitors [9]. Over this interval fasting blood glucose levels rise in parallel with the worsening heart failure. We previously reported that administration of the GLUT4 antagonist ritonavir to 75 day old TG9 mice acutely exacerbates glucose intolerance and precipitates decompensated heart failure [10]. This model therefore provides a useful system to investigate the influence of improved glucose homeostasis on myocardial function and survival.

**Figure 1:**
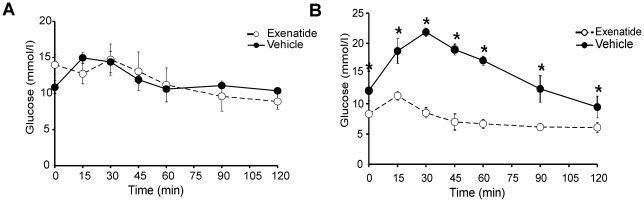
Intraperitoneal glucose tolerance tests (1 g/kg) on age-matched female TG9 mice following a 5 hour fast. **A**. Baseline responses at 8 weeks of age. **B**. Following 2 weeks of treatment with either vehicle or exenatide (Age 10 weeks). Data is shown as the mean ± SEM (n = 4-6). *p<0.01, ANOVA.

**Figure 2:**
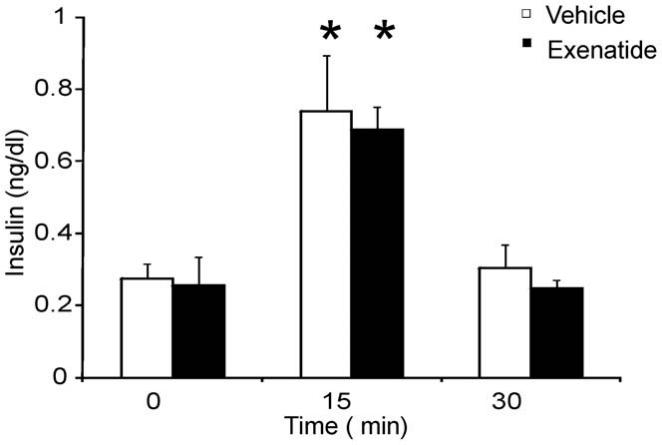
Insulin levels were obtained on age matched female TG9 mice at 10 weeks following a 5 hour fast at 0, 15 and 30 minutes after an intraperitoneal glucose dose (1 g/kg). Data shown as the mean ± SEM (n = 4 per group). *, p<0.05 compared to 0 minute insulin level.

To determine the effect of exenatide on glucose homeostasis, female TG9 mice fed a standard chow diet first underwent intraperitoneal glucose tolerance tests at 8 weeks of age. At this age, TG9 mice appear overtly normal and have normal fasting blood glucose levels but have echocardiographic evidence of mild dilated cardiomyopathy [10]. Glucose tolerance tests (GTT) were repeated after administering exenatide subcutaneously (40 µg/kg/day divided into two daily doses) or vehicle for 14 days. Baseline blood glucose levels and responses to GTT did not differ between the two treatment groups (Figure 1). At 10 weeks of age, TG9 mice receiving exenatide exhibited a response to glucose challenge that was similar to untreated non-transgenic littermates with fasting and peak blood glucose levels of 6.9±0.3 and 11.3±0.5 mmol/L, respectively. In contrast, vehicle-treated TG9 mice developed a significant worsening in both fasting blood glucose levels (12.2±1.7 mmol/L) and glucose tolerance (peak glucose 21.8±0.7 mmol/L). None of the mice experienced detectable hypoglycemia at any point during the study. As shown in Figure 2, there was no difference in fasting serum insulin levels in 10 week old exenatide-treated animals (0.26±0.08 ng/ml) compared to vehicle-treated littermate controls (0.27±0.04 ng/ml). There was also no difference in insulin levels 15 minutes (0.69±0.06 ng/ml exenatide vs 0.74±0.15 ng/ml vehicle) or 30 minutes (0.25±0.02 ng/ml exenatide vs 0.30±0.06 ng/ml vehicle) after a 1 g/kg intraperitoneal glucose load. Thus, exenatide appears to normalize the response to glucose challenge in treated TG9 animals by increasing insulin sensitivity rather than insulin secretion. Exenatide did not alter lipid profiles in TG9 mice (Table 1).

**Table 1:** Lipid profile of TG9 mice.

Parameter	Exenatide (n = 4)	Vehicle (n = 4)	p-value
Triglycerides (mg/dl)	135.0±22.5	140.9±29.78	0.88
Cholesterol (mg/dl)Free	106.5±8.47	103.4±6.88	0.79
Free Fatty Acids (mM)	0.97±0.31	0.61±0.13	0.32

Lipid profiles were measured in plasma of mice at 70 days of life. Data is shown as average +/− SEM. P-values determined by unpaired student's t-test.

### Glucose transporter expression and function

**Figure 3:**
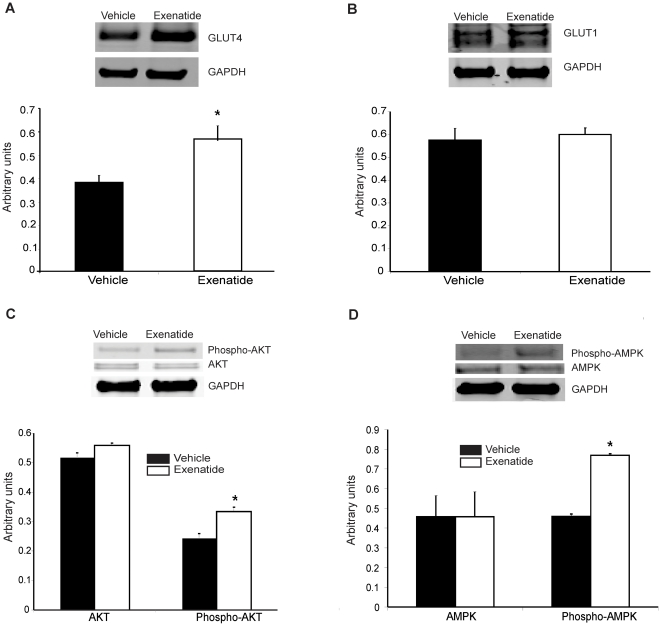
Western blot analysis and protein quantification of left ventricular myocardium harvested from 70-day-old TG9 mice. **A**. GLUT4 **B**. GLUT1 **C**. pan-AKT and phospho-AKT. **D**. AMPKα and phospho-AMPK. For each protein, the top panel represents expression as determined using antibody recognizing the protein of interest. Bottom panel: Data is represented as the mean protein intensity normalized to GAPDH from 4–6 independent mice. Each western blot was performed in triplicate. Values are expressed as the mean ± SEM; * indicates p<0.03, student's t-test.

While the diabetic heart is known to use fatty acids almost exclusively as a fuel [14], [15], glucose utilization is increased in hypertrophic cardiomyopathy despite the development of insulin resistance [16]. This is accompanied by increased expression of the constitutively active glucose transporter GLUT1 [17]. In TG9 mice GLUT1 levels are similarly increased whereas the expression of GLUT4, the predominant glucose transporter in the adult heart, is unchanged [10]. We therefore examined whether GLUT expression was altered by exenatide in TG9 mice. At 75 days of age, total myocardial GLUT4 protein levels were increased by 40% in exenatide-treated versus non-treated TG9 mice whereas GLUT1 protein levels were unchanged (Figure 3A, B). Although previous studies have shown that exenatide can increase the expression of GLUT1, which is constitutively present on plasma membranes of multiple tissues including the heart and skeletal muscle [18], we did not detect any differences in total GLUT1 levels possibly because heart failure alone induces maximal up-regulation of this transporter [10].

Measurement of total AKT revealed no difference between the 2 groups at 70 days (Figure 3C). Phosphorylated AKT, however, was approximately 40% higher in the exenatide-treated animals. Similarly, while AMPKα levels were unchanged, phosphorylated AMPKα was increased by 67% in the exenatide versus vehicle-treated mice (Figure 3D).

**Figure 4:**
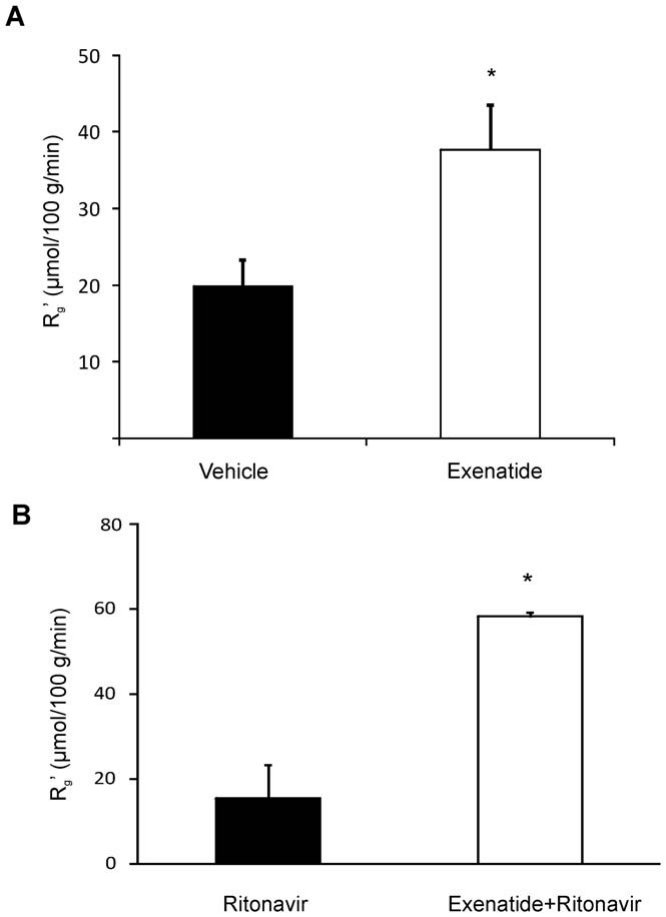
Left ventricular myocardial glucose uptake in 75-day old female TG9 mice. **A**. TG9 mice treated with vehicle or exenatide starting at 56 days of age as determined by [^3^H]-2-deoxyglucose incorporation following a 5-h fast (n = 9 and 8 per group). **B**. TG9 mice treated with vehicle or exenatide as in panel A were treated with ritonavir (10 mg/kg) administered by i.p. injection 15 minutes prior to measurement of [^3^H]-2-deoxyglucose incorporation (n = 6 and 3 respectively per group). Tissue glucose metabolic index (Rg'); *p<0.05.

We next determined whether exenatide affects relative cardiac glucose uptake in TG9 mice. We have previously demonstrated that in 75 day old TG9 mice 2-deoxyglucose (2-DG) uptake is reduced to approximately one third of that observed in non-transgenic littermate control animals, consistent with the development of insulin resistance in these animals [10]. Acute treatment of 56 day old TG-9 mice with exenatide did not alter basal 2-deoxyglucose uptake into left ventricular myocardium (10±3.8 µmol/100 g/min) compared to vehicle-treated animals (14.5±8.3 µmol/100 g/min). However, as shown in Figure 4A, after 3 weeks of exenatide treatment, 2-DG uptake was significantly increased (37.7±5.9 µmol/100 g/min) compared to vehicle treated animals (19.8±3.6 µmol/100 g/min, p<0.05). Under these fasting conditions, basal skeletal muscle 2-DG uptake was low and no significant change was detected (data not shown).

We have previously shown that acute administration of the GLUT4 antagonist ritonavir produces a significant reduction in myocardial glucose uptake in TG9 mice [10]. Given that chronic exenatide treatment enhanced myocardial GLUT4 expression and glucose uptake, we reasoned that this GLP-1 agonist should ameliorate the detrimental effect of ritonavir. TG9 mice were chronically treated with exenatide or vehicle starting at 56 days of life and then exposed to a single dose of intraperitoneal ritonavir (10 mg/kg) 15 minutes prior to tail vein injection of 2-deoxyglucose (2-DG). As shown in Figure 4B, myocardial glucose uptake was significantly increased (58.3±1.0 µmol/100 g/min) in ritonavir-treated mice with chronic prior exenatide exposure compared to animals that had received vehicle over this same interval (15.4±7.9 µmol/100 g/min, p = 0.002).

### Cardiac function

The effect of exenatide on cardiac contractile function was assessed by echocardiography in 70 day-old TG9 mice. It was noted that the vehicle-treated mice were hypothermic and bradycardic compared to exenatide-treated animals (Table 1). Bradycardia is commonly observed in rodents with severe heart failure and is thought to be a compensatory mechanism to increase left ventricular filling time in mice with normally fast heart rates [19]. Upon warming the control animals to normothermia, heart rates remained lower but this difference did not reach statistical significance. Systolic function, as assessed by fractional shortening was significantly improved in the exenatide-treated mice. Left ventricular remodeling, as assessed by the left ventricular diameter and posterior wall thickness in end-diastole, was similar in the treated and untreated animals (Table 2).

**Table 2:** Echocardiography of TG9 mice.

Parameter	Exenatide (n = 6)	Vehicle (n = 8)	p-value
Body weight (g)			
Age 50 days	21.7±0.38	20.2±0.42	0.51
Age 70 days	21.9±0.31	20.9±0.5	0.23
Heart rate (beats per minute)	491±19	466±14	0.34
Basal body Temperature (°C)	36.4±0.1	34.7±0.2	0.014
LVPWd	0.70±0.05	0.73±0.02	0.66
LVIDs	2.81±0.12	3.17±0.12	0.048
LVID_d_	4.00±0.13	3.98±0.12	0.98
Fractional shortening (%)	26.8±1.4	20.7±1.8	0.03

Transthoracic echocardiography performed on 70 day old TG9 mice. Exenatide (or vehicle) was administered subcutaneously at a dose of 40 µg/kg/day (divided b.i.d.) starting at 56 days of age. Hypothermic animals were warmed to 36°C immediately prior to obtaining echocardiograms. LVPWd, left ventricular posterior wall diastolic dimension. LVIDs, left ventricular internal diameter in systole. LVIDd, left ventricular internal diameter in diastole. Data is shown as the average +/− SEM.

**Figure 5:**
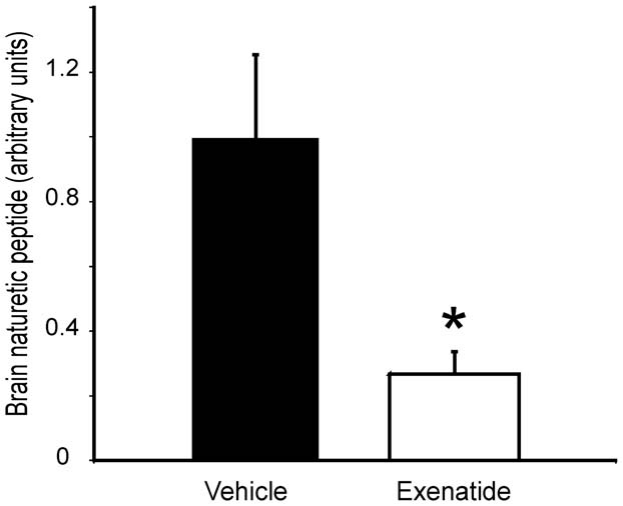
RT- PCR for brain naturetic peptide measurement in the TG9 left ventricular tissue harvested at 70 days of life. Mice were treated with exenatide or vehicle (40 µg/kg/day divided b.i.d.) starting at 56 days. n = 4 per group, p-value  = 0.02.

For an independent marker of the severity of heart failure, we measured mRNA expression of brain natriuretic peptide (BNP) in the left ventricle of exenatide-treated mice and the control counterparts. As shown in Figure 5, BNP levels were significantly lower in the exenatide-treated animals compared to littermate vehicle-treated control TG9 mice (0.28±0.07 versus 1.0±0.26 arbitrary units, respectively, p = 0.02).

### Survival analysis

**Figure 6:**
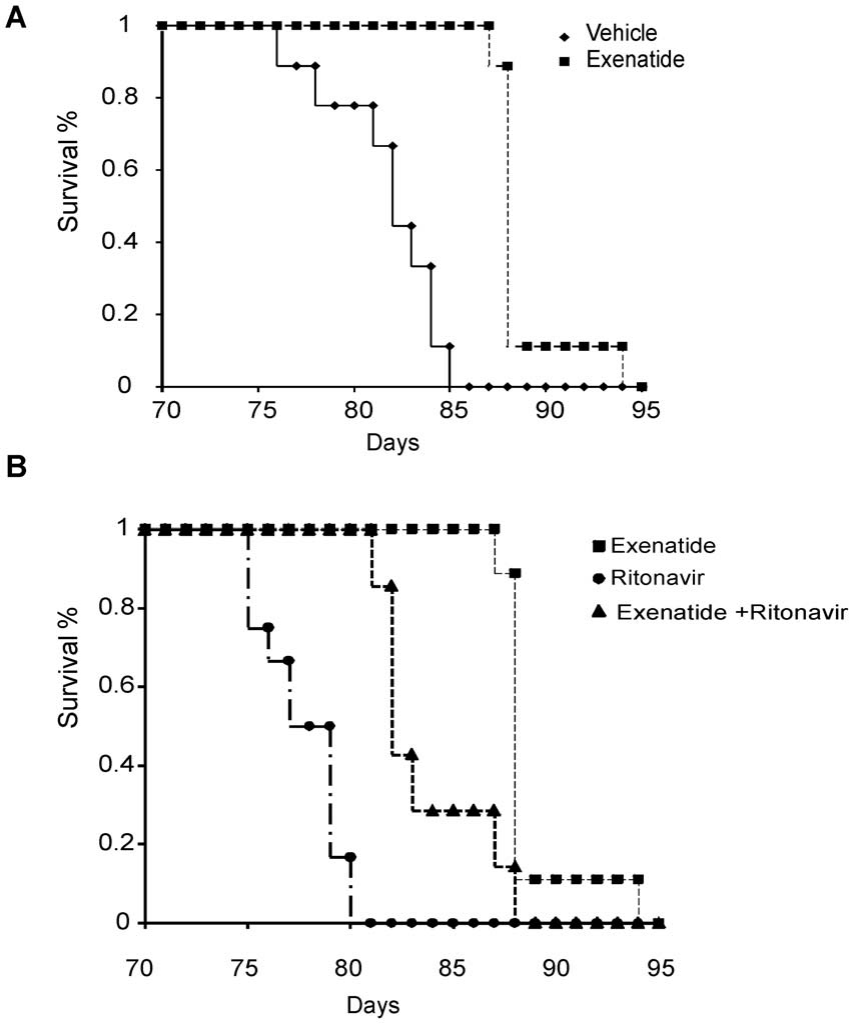
Kaplan-Meier survival curves of female TG9 mice treated with 40 µg/kg/day of exenatide subcutaneously (divided b.i.d.) or vehicle beginning at 56 days of life and continuing until the time of death. Panel A: Exenatide-treated mice (n = 9) versus vehicle (n = 6), p-value <0.01. Panel B: Exenatide and vehicle treated mice given ritonavir daily by intraperitoneal injection (10 mg/kg) beginning at 75 days of age and continuing until the time of death (n = 7 and 12 respectively per group).

Although various genetic and pharmacologic interventions can improve markers of cardiac function in heart failure, prolongation of survival is the gold standard outcome. The highly reproducible progression of heart failure and death in the TG9 mice provides a means to assess survival as an endpoint. Untreated TG9 mice typically die between 11–13 weeks of age (mean 81±2 days for females). In addition to improving glucose tolerance and cardiac function, exenatide significantly improved survival of TG9 mice compared to vehicle-treated littermate controls (Figure 6A) (89.6±2 days vs. 83.0±0.6 days). This effect is comparable to the improvement afforded by the ACE inhibitor captopril [9].

Consistent with our previous study [10], intraperitoneal injection of ritonavir starting at 75 days of age produced immediate signs of decompensated heart failure including decreased activity and tachypnea (data not shown). The decompensation was transient, consistent with the pharmacokinetics of drug elimination [20], but sometimes caused death. As shown in Figure 6B, when ritonavir administration was continued daily, survival was significantly reduced (78.6±0.6 days, p<0.05). Mice treated with both ritonavir and exenatide survived a mean of 84.6±1.0 days, which is significantly longer than mice given ritonavir alone and is close to the survival age of TG9 mice given vehicle alone. This further supports a significant influence of glucose transport on overall survival in this rodent model.

## Discussion

Maladaptive neurohormonal signaling involving the renin-angiotensin-aldosterone and adrenergic systems are fundamental pathophysiological processes in heart failure. Insulin resistance and abnormal myocardial glucose uptake also exist, but whether they are adaptive or not is less clear. The present analyses of pharmacologic manipulations in an animal model of dilated cardiomyopathy indicate that the metabolic changes are maladaptive. Exenatide, an incretin mimetic, improves glucose homeostasis, delays the progression of heart failure and most importantly prolongs survival. This study extends previous reports showing that GLP-1 has beneficial short-term effects on cardiac contractility and cardiac output in heart failure patients [7] and provides further support for a causal link between changes in glucose uptake and cardiac function. Our data are also consistent with the increased survival observed by Poornima and colleagues in heart failure prone rats given a 3-month continuous infusion of GLP-1 [21]. In their chronically hypertensive rat model, heart failure occurs secondarily to the metabolic syndrome phenotype. In contrast, in the TG9 mouse heart failure is a primary effect of a cardiac-specific transgene. Therefore, we demonstrate that treatment of insulin resistance arising secondarily from a primary cardiomyopathy is beneficial.

Complex factors involving the heart, other organs or systems and their interactions clearly influence survival in heart failure. The beneficial effects of exanatide on cardiac function and survival may therefore be mediated through drug-induced changes in the heart, the body, or both. Exenatide and ritonavir can have different influences on individual factors; their combined effects determine the pathophysiologic variables related to glucose homeostasis, contractile function or survival. Thus, while exenatide can normalize whole-body insulin sensitivity and myocardial glucose uptake in mice that have advanced dilated cardiomyopathy, cardiac contractile function and survival are improved but not to perfect health. Exenatide in the failing heart can also abrogate the inhibitory effect of ritonavir on myocardial glucose uptake. However, while protecting against death from acute cardiac decompensation precipitated by ritonavir, exenatide does not completely prevent it, as shown by the small but significant difference in survival between animals given exenatide and ritonavir or exenatide alone. Ritonavir may therefore have extracardiac effects not completely rescued by exenatide treatment, e.g., impaired respiratory mechanics in the setting of acute pulmonary congestion, or less likely unknown cardiac effects unrelated to myocardial glucose transport.

Although exenatide clearly has complex effects in heart failure, the expression of the G-protein-coupled receptor GLP-1R in cardiomyocytes supports the role of a direct effect on the heart. Studies in GLP-1R^−/−^ mice indicate that signaling through this receptor affects both heart rate and contractile function [22]. Furthermore, native GLP-1 has been shown to improve cardiac reconditioning in isolated perfused rat hearts [23]. Signaling through GLP-1 could facilitate activation of AKT or AMPK signaling and consequent GLUT4 translocation.

Since insulin-stimulated activation of AKT leads to GLUT4 translocation, the increased levels of phosphorylated AKT observed in exenatide treated mice likely contributes to increased myocardial glucose uptake. The augmented glucose uptake observed in our study can also be mediated through AMPKα activation. AMPK is a key molecular player in energy homeostasis at both cellular and whole-body levels [25]. In the heart, AMPK activation is known to increase cardiac glucose utilization by a number of different mechanisms including the translocation of GLUT4 to the sarcolemma, with a resulting augmentation of glucose uptake [26]. Metformin, which activates AMPK similarly prevents the progression of heart failure in dogs [27]. It remains to be determined whether this AMPK activation is mediated directly through the myocardial GLP-1 receptor or indirectly through improved contractile function. Investigation of the effects of exenatide in GLP-1R^−/−^ and GLUT4^−/−^ mice will provide further insight into direct versus indirect drug effects. Exenatide could also have effects in chronic heart failure that are unrelated to glucose-transport such as the inhibition of apoptosis [24].

Ideally, any novel heart failure therapy, whether it inhibits a maladaptive or supplements an adaptive response, should be free of serious side-effects. GLP-1 agonists, which are relatively new agents in the treatment of type 2 diabetes, possess several benefits over other therapies including less risk for hypoglycemia, modulation of satiety with less weight gain, and preservation of beta cell mass [6]. The pharmacokinetic profile of exenatide allows intermittent subcutaneous dosing rather than continuous infusion as is required of GLP-1. However, exenatide may be linked to the development of pancreatitis [6], and the potential for interactions with other drugs commonly prescribed in heart failure patients exists. Thus continued research into and development of incretin-based therapies that mimic GLP-1 or prevent its degradation are worthwhile.

Whether incretin-based drugs like exenatide or dipeptidyl peptidase inhibitors that augment endogenous GLP-1 levels can provide an additional benefit when used in combination with standard heart failure therapies is an open question. The current findings may also have physiologic relevance to other situations such as the ischemic heart, which loses its normal substrate flexibility and becomes more dependent upon glucose as a metabolic fuel [28]. Finally, the ultimate question is whether the survival benefit of exenatide in a murine model will translate to human patients. We suggest that the strong conservation of molecular pathways in mouse and human cardiovascular physiology provides a compelling rationale to address this question.
